# Concurrent infections of dengue virus serotypes in Bali, Indonesia

**DOI:** 10.1186/s13104-019-4164-9

**Published:** 2019-03-12

**Authors:** Sri Masyeni, Benediktus Yohan, R. Tedjo Sasmono

**Affiliations:** 1 Faculty of Medicine and Health Sciences, Warmadewa University, Jl. Terompong No. 24, Denpasar, Bali 80235 Indonesia; 2 Eijkman Institute for Molecular Biology, Ministry of Research, Technology, and Higher Education of the Republic of Indonesia, Jl. Diponegoro 69, Jakarta, 10430 Indonesia

**Keywords:** Dengue, Concurrent infections, Serotype, Bali, Traveler

## Abstract

**Objective:**

To describe cases of dengue virus (DENV) concurrent infections in patients from both local and international traveler visiting Bali, Indonesia.

**Results:**

During a hospital-based study, 260 patients (from 161 local and 99 international traveler patients) were recruited. Among them, 190 were positive by DENV RT-PCR in which eight patients (five local and three international travelers) detected as having concurrent infections by two different DENV serotypes. Among the eight patients, the common dengue symptoms diagnosed were fever, headache, and myalgia. Six cases (75%) were diagnosed with dengue fever (DF) while two cases (25%) manifested with bleeding and were diagnosed with dengue hemorrhagic fever (DHF) grade 1. The DENVs concurrent infections involved all four DENV serotypes known to be circulating in Bali. Although cases of DENV concurrent infections have been implicated with severe manifestation, we observed that most of concurrent infections cases in our study were of mild clinical manifestation, that may be related to the changing of DENV serotype predominance which is occurring in Bali, Indonesia.

## Introduction

The dengue disease, caused by the etiological agent dengue virus (DENV) has been considered as the most important arboviral infection in the world, with significant burden [1]. All four serotypes of DENV (DENV-1, -2, -3, and -4), members of the *Flaviviridae* family, may induce long-lived serotype-specific immunity and only confers short-lived cross-immunity [2]. Clinical manifestations of DENV infection vary from asymptomatic, mild dengue fever (DF), to the more severe dengue hemorrhagic fever (DHF) and life-threatening dengue shock syndrome (DSS), for particular people [3–5]. Risk factors for severe dengue are young age, female sex, high body-mass index, virus strain, and genetic variants [6–8].

All four DENV serotypes were found to circulate in Asia, Africa and America with co-circulation of multiple DENV serotypes reported [9]. Co-circulation of DENV serotypes increases the risk of concurrent infections, a phenomenon best observed in epidemics [10, 11]. It has been reported that the occurrence of concurrent infections was ranging from 5% to as high as 50% [11].

Conflicting outcomes were reported in which concurrent infections of DENV serotypes may lead to mild or severe dengue manifestation [11–15]. The concurrent infections of DENV-serotypes in Indonesia has been reported, including in Bali [16] which has been known as a famous tourist destination both local and international. The hyperendemicity of DENV in Bali may affect both local people and international travelers [17].

In this report, we present cases of DENV concurrent infections in local Balinese and international travelers admitted to tertiary hospitals in Bali. The clinical manifestation characteristics related to cases of DENV concurrent infections were sought and assessed together with molecular surveillance data.

## Main text

### Methods

#### Sample collection and processing

This study was part of dengue and other viral diseases molecular surveillance study in local and international travelers visiting Bali, commenced in 2015–2017 [16, 17]. Samples were collected from patients seeking medication to three tertiary hospitals in Bali, namely RS Kasih Ibu, Denpasar; RS Sanjiwani, Gianyar; and RS Wangaya, Denpasar. Serum samples were obtained from patients’ blood during acute phase from day 0 up to day 5 of fever onset and convalescence sera were taken on patients’ hospital discharge day. All patients were adults with age of > 14 years old. The grading of dengue clinical manifestation was implementing the WHO-SEARO guidelines [8] and diagnosis of dengue hemorrhagic fever (DHF) was based on the evidence of plasma leakage which includes the hematocrit (HCT) level increase or the findings of pleural effusion or ascites on physical examination. The assessment of plasma leakage evidence was performed based on > 20% drop in HCT following volume replacement treatment compared to baseline [8], i.e. HCT level differences of more than 20% between the highest HCT level recorded during the phase of illness and level during convalescence. Thrombocytopenia was defined as a rapid decline of platelet with platelet count of < 100 × 10^9^/L.

#### Serology testing, DENV detection, serotyping, and genotyping

The anti-DENV IgM and IgG serology detection was done using Panbio Dengue Duo IgM/IgG ELISA kit (Alere, Brisbane, Australia) to determine the infection status as primary or secondary infection, as described previously [18]. Dengue was confirmed through DENV NS1 antigen detection using the SD Bioline NS1 Rapid Test (Alere). The DENV RNA was extracted from serum sample using QIAamp Viral RNA Mini kit (Qiagen, Hilden, Germany) according to the manufacturer’s instructions, and used as template in simultaneous DENV detection and serotyping by real-time RT-PCR using Simplexa Dengue (DiaSorin, Cypress, CA, USA), as described elsewhere [19]. The positively detected DENV nucleic acid in samples was reported as the resulting DENV serotype and its Ct value correspond to the semi-quantitative measurement of viral titer where lower Ct value correlate with higher viral titer and vice versa. Genotype determination was performed using Sanger capillary sequencing of the DENV envelope (E) gene, as described elsewhere [20].

### Results

#### Clinical and laboratory examinations of the concurrent infections cases

A total of 260 samples were collected during the study. The total samples comprise of 161 local and 99 international traveler patients. Among them, eight patients were of DENV concurrent infections as simultaneously detected and serotyped using real-time RT-PCR. The clinical and laboratory examinations data of patients with DENV concurrent infections were summarized in Table 1.

**Table 1:** Demographic and clinical variables among concurrent infections cases

Variable	Case 1	Case 2	Case 3	Case 4	Case 5	Case 6	Case 7	Case 8
*Demography and clinical manifestations*								
Age range, years old	20–30	20–30	30–40	40–50	30–40	40–50	50–60	50–60
Race/nationality	Balinese	Balinese	Balinese	Balinese	Balinese	French	French	Austrian
Gender	Male	Female	Male	Male	Male	Female	Male	Male
Fever day	3	3	3	5	2	3	3	3
Headache	+	+	+	–	–	+	+	+
Arthralgia	+	+	+	+	–	+	–	+
Myalgia	+	+	+	+	–	+	–	+
Vomiting	+	+	–	–	–	+	–	–
Bleeding manifestation	+ (Petechiae)	–	–	–	–	+ (Gum)	–	–
Shock	–	–	–	–	–	–	–	–
Plasma leakage	–	+	–	+	–	–	–	–
Comorbidity	–	–	–	–	–	–	+ (DVT)	–
*Laboratory examinations data*								
Nadir WBC (× 10 µg/µL)	4.10	3.00	2.70	2.90	2.06	1.16	2.84	1.13
HCT difference (%)	12.0	21.4	9.9	21	11.9	6.1	7.4	11.1
Nadir PLT (× 10^9^/L)	122	91	16	56	114	56	117	113
AST (U/L)	34	167	N/A	164	N/A	24	59	40
ALT (U/L)	23	190	N/A	168	N/A	14	42	36
Albumin (g/L)	3.8	3.0	N/A	3.4	N/A	N/A	3.8	3.8
DENV NS1	+	+	+	+	+	+	+	+
Dengue IgM	+	+	–	+	+	+	+	+
Dengue IgG	+	+	+	+	+	–	–	–
Infection status	Secondary	Secondary	Secondary	Secondary	Secondary	Primary	Primary	Primary
Severity	DF	DHF grade 1	DF	DHF grade 1	DF	DF	DF	DF
DENV Serotype (RT-PCR Ct value)^a^	DENV-2 (19.8), DENV-4 (38.7)	DENV-1 (16.0), DENV-2 (35.1)	DENV-1 (16.3), DENV-3 (29.2)	DENV-1 (33.3), DENV-3 (31.9)	DENV-3 (29.0), DENV-4 (38.5)	DENV-1 (29.1), DENV-2 (29.1)	DENV-1 (25.2), DENV-2 (20.6)	DENV-2 (24.5), DENV-4 (22.7)

+, positive; −, negative; DF, dengue fever; DHF, dengue hemorrhagic fever; WBC, white blood cell; HCT, hematocrit; PLT, platelet; AST, aspartate aminotransaminase; ALT, alanine transaminase; DVT, deep vein thrombosis; N/A, not applicable due to insufficient sample volume

^a^Determined using Simplexa Dengue qRT-PCR

All cases presented with fever and the majority of patients had headache, myalgia, and vomiting. There was no serious bleeding observed in cases, except for petechiae and gum bleeding. Likewise, there was no fluid accumulation or shock observed in all cases. The level of liver enzymes and albumin were gradually reached the relatively normal level. Laboratory results showed no signs of leukocytosis in majority of the cases with relatively normal hemoglobin levels. Thrombocytopenia was observed only in case 2, 3, 4, and 6.

#### DENV infection status, serotypes, and genotypes

In term of DENV infection status as inferred from serology results, most of the cases were of secondary infection. We observed that secondary infection was observed in all local patients, while all international travelers were characterized as of primary infection (Table 1). Serology detection revealed the IgM positivity of 87.5% among concurrent infections cases.

From 260 collected samples, 190 were successfully determined for their infecting DENV serotype. Among them, 182 samples were of mono-infection while 8 samples were of concurrent infections of two different DENV serotypes. The DENV concurrent infections involved all four DENV serotypes with most of the cases involving DENV-1 (Table 1). Three cases were of DENV-1 and -2 concurrent infections while two cases involving DENV-1 and -3. The remaining two cases involved DENV-2 and -4 and one case serotyped as having DENV-3 and -4. In term of the Ct values of each DENV serotype measured using real-time RT-PCR, majority of cases showed relatively equal Ct values although there was presence of higher Ct value in one serotype than the other serotype of the concurrent infections (Table 1).

Genotype determination was performed to one case of concurrent infections (Case 8) where both of the DENV-2 and -4 serotypes were successfully sequenced (data not shown). Utilizing Twiddy, et al. classification for DENV-2 [21], the DENV-2 isolate of case 8 was grouped into the Cosmopolitan genotype. The other DENV-4 isolate of case 8 was classified into the Genotype II of DENV-4 grouping based on Lanciotti, et al. classification [22].

#### Data comparison between DENV mono-infection and concurrent infections cases

We performed a comparison between clinical and laboratory data from cases with mono-infection and concurrent infections (Table 2). However, the small number of concurrent infections cases was limiting statistical analysis. Despite the gender proportion, other clinical and laboratory experiment data showed similar features, including the infection status where the majority of mono-infection and concurrent infection cases were of secondary infection. In term of severity, concurrent infection cases showed relatively milder manifestations than cases of mono-infection.

**Table 2:** Demography, clinical, and laboratory data comparison between mono-infection and concurrent infections cases during dengue surveillance in Bali

Variable	Mono-infection cases (N = 182)	Concurrent infections cases (N = 8)
*Demography and clinical manifestations*		
Age, median year (range)	28 (14–75)	42 (25–53)
Male, N (%)	80 (44.0)	7 (87.5)
Female, N (%)	102 (56.0)	1 (12.5)
Fever day, days ± SD	3.3 ± 0.83	2.0 ± 0.93
Headache	139 (76.4)	7 (87.5)
Arthralgia	101 (55.5)	6 (75.0)
Myalgia	122 (67.0)	6 (75.0)
Vomiting	92 (50.5)	2 (25.0
Bleeding	46 (25.3)	2 (25.0)
Plasma leakage	0 (0.0)	0 (0.0)
Shock	0 (0.0)	0 (0.0)
*Laboratory examinations data*		
Nadir WBC (× 10 µg/µL), median (range)	2.1 (0.48–6.4)	2.77 (1.16–4.1)
HCT difference (%), median (range)	13.1 (2.43–68.9)	11.5 (6.1–21.4)
Nadir PLT (× 10^9^/L), median (range)	43 (6–194)	102 (16–122)
DENV NS1 test positivity, N (%)	173 (95.1)	8 (100.0)
Dengue IgM positivity, N (%)	123 (67.6)	7 (87.5)
Dengue IgG positivity, N (%)	142 (78.0)	5 (62.5)
*Dengue severity*		
DF, N (%)	110 (60.4)	6 (75.0)
DHF grade 1, N (%)	42 (23.1)	2 (25.0)
DHF grade 2, N (%)	26 (14.3)	0 (0.0)
DHF grade 3, N (%)	4 (2.2)	0 (0.0)
DHF grade 4, N (%)	0 (0.0)	0 (0.0)

DF, dengue fever; DHF, dengue hemorrhagic fever; WBC, white blood cell; HCT, hematocrit; PLT, platelet

### Discussion

The circulation of multiple DENV serotypes within the same geographical area increases the risk of concurrent infections that mostly occurs during an epidemic [10, 11]. The first case of DENV concurrent infections was reported from a study in Puerto Rico in 1982 [14]. Historically, DENV concurrent infections cases has been reported in samples isolated in Indonesia since 1976 [15]. The recent surveillance reports from Indonesia reported the DENV concurrent infections occurrence ranged from 1.6 to 29.0% [16, 18, 20, 23, 24].

It has been proposed that DENV hyper-endemicity, which leads to concurrent infections, was the contributing factor for severe dengue [12]. However, in this case report, the occurrence of DENV concurrent infections in patients was related to relatively mild clinical manifestation with no serious bleeding, fluid accumulation or shock observed (Table 1). The clinical assessment results were supported with laboratory examination data in which no signs of leukocytosis, one of the indicators of severe dengue [25], was detected. However, signs of thrombocytopenia were observed in some of the patients (Table 1). Two cases were clinically graded as DHF grade I due to the HCT levels difference of more than 20% between acute and convalescence sera (Table 1).

The majority of concurrent infections cases were of secondary infection as determined by serology and all of them are patients from local Balinese. It has been reported that the number of secondary infection in adults are higher, especially in an endemic area like Bali with prolonged exposure to dengue infection in the past [16]. Interestingly, cases from international travelers were of primary infection with only anti-DENV IgM antibody detected (Table 1). Looking into their history, the travelers stated that they have never been contracted to dengue in the past. Moreover, the travelers were coming from countries where dengue is not endemic.

The DENV serotypes involved in concurrent infections were mostly involved DENV-1 and dominated by mix of DENV-1 and -2, followed by DENV-1 and -3, DENV-2 and -4, and DENV-3 and -4. In term of clinical manifestation caused by DENV concurrent infections, our findings are in line with previous reports from Brazil which observed mild dengue manifestation caused by concurrent infections of DENV-1 and DENV-2 [26, 27] and DENV-3 and DENV-4 [28]. The involvement of DENV-1 in majority of the concurrent infections cases was also observed in previous reports of concurrent infections in Semarang [20] and Surabaya [18]. Reports have been proposing the phenomena of serotype replacement in places in Indonesia where DENV-1 has becoming the predominant serotype [18, 20, 23, 24, 29]. Our previous dengue surveillance in local Balinese observed the rise of DENV-1 infection as the second most common serotype detected in patients [16]. It may happen that this cyclical serotype predominance is currently occurring in Bali, the condition that needs to be continuously monitored. The relationship of DENV-1 predominance with mild dengue manifestation has also been observed in a surveillance study in Jambi, Sumatra [23].

Previous studies have correlated the higher viremia level with severe dengue clinical manifestation in DENV concurrent infections [12, 30]. In a case report, significantly higher titer of DENV-3 over DENV-2 was recorded in which may suggest the better replication rate of the former compared to the latter [12]. However, in this study, only one DHF case was reported to have significantly higher viral titer (as of Ct value) of DENV-1 over DENV-2. In other DHF case, similar Ct values were recorded. Moreover, we did not observe the correlation of higher DENV serotype titer with severe clinical manifestation in the majority of cases (Table 1). It has also been reported that the host (secondary infection status) and viral factors (viral load titer) influenced the clinical phenotype of severe dengue manifestations [31]. Our data may suggest that there may be other factors influencing clinical manifestation, especially in concurrent infections cases.

Looking deeper into the genotypes of the DENV serotypes from the concurrent infections cases, both genotypes of the infecting DENV serotypes were successfully obtained for one case. The DENV-2 isolate of case 8 were grouped into Cosmopolitan genotype and the DENV-4 classified as Genotype II. These classifications were still correspond to the genotype data of DENV in Bali [16], reflecting the endemicity of DENV and occurrence of active virus transmission in the island.

In term of comparison between concurrent infection samples and other samples detected as having only single or mono infection, we observed a relatively similar clinical and laboratory profiles where relatively milder manifestations were observed in concurrent infections cases (Table 2). Nevertheless, both groups reflected mild dengue manifestations where the majority of them were of DF manifestation, with only a small proportion developing DHF [16, 17]. Although it has been reported that co-infected patients are skewed towards more severe clinical manifestations compared to mono-infected patients [11], in this research note, our results suggested that patients having concurrent infections of DENV were not prone to severe disease.

In conclusions, we report cases of DENV concurrent infections among local Balinese and international travelers. We highlighted that the concurrent infections were not directly associated with severe dengue.

## Limitations

The small number of concurrent infections samples described may not be sufficient to describe the clinical manifestation related to DENV concurrent infections. Hence, this finding needs to be supported with bigger and nation-wide surveillance data.

## Abbreviations

DENV: dengue virus
DF: dengue fever
DHF: dengue hemorrhagic fever
DSS: dengue shock syndrome
RT-PCR: reverse transcriptase polymerase chain reaction
NS1: non-structural protein 1
RNA: ribonucleic acid
HCT: hematocrit
IgM: immunoglobulin M
IgG: immunoglobulin G
PLT: platelet
AST: aspartate aminotransaminase
ALT: alanine transaminase
DVT: deep vein thrombosis
